# Retraction: The adoption of renewable energy towards environmental sustainability: Evidence from Partial Least Square Structural Equation Modelling (PLS-SEM)

**DOI:** 10.1371/journal.pone.0333980

**Published:** 2025-10-09

**Authors:** The PLOS One Editors

The *PLOS One* Editors retract this article [1] because it was identified as one of a series of submissions for which we have concerns about potential manipulation of the publication process, peer review integrity, and authorship. These concerns call into question the validity and provenance of the reported results. We regret that the issues were not identified prior to the article’s publication.

JG and SB did not agree with the retraction. XY and GW either did not respond directly or could not be reached.

## References

[1] Gyimah J, Batasuma S, Yao X, Wauk G. The adoption of renewable energy towards environmental sustainability: Evidence from Partial Least Square Structural Equation Modelling (PLS-SEM). PLoS One. 2024;19(4):e0299727. doi: 10.1371/journal.pone.0299727 38573973 PMC10994344

